# From zoster to polyneuropathy: manifestations, mechanisms, and differential diagnosis of cranial nerve involvement in Ramsay Hunt Syndrome

**DOI:** 10.3389/fneur.2026.1732724

**Published:** 2026-04-09

**Authors:** Shusheng Jiao

**Affiliations:** Department of Neurology, Bethune International Peace Hospital, Shijiazhuang, Hebei, China

**Keywords:** cranial nerve, facial nerve, polycranial neuropathies, Ramsay Hunt Syndrome (RHS), varicella-zoster virus (VZV)

## Abstract

Ramsay Hunt Syndrome (RHS) arises from reactivation of varicella-zoster virus (VZV) in the geniculate ganglion, clinically manifesting as facial palsy and an auricular rash. Involvement of cranial nerves (CN) beyond the facial nerve (CN VII) occurs in up to half of cases, complicating the clinical presentation. Current knowledge regarding polycranial involvement in RHS remains fragmented, largely drawn from isolated case reports and small series. This review synthesizes available evidence on the epidemiology, clinical spectrum, differential diagnosis, and mechanisms of cranial nerve involvement in RHS. Due to anatomical proximity, the vestibulocochlear nerve (CN VIII) is most frequently affected. Involvement of other cranial nerves—including the trigeminal (CN V), glossopharyngeal/vagus (CN IX/X), ocular motor nerves (CN III, IV, VI), and rarely the accessory (CN XI) or hypoglossal (CN XII) nerves—whether isolated or combined, typically reflects more extensive disease and is associated with a poorer prognosis. Advanced age, immunocompromised status, and delayed initiation of antiviral and corticosteroid therapy further influence clinical outcomes. Pathogenesis involves both direct axonal and hematogenous spread of VZV, likely accompanied by immune- and inflammatory-mediated injury. The diversity of polycranial presentations necessitates careful differential diagnosis. Future efforts should focus on elucidating molecular mechanisms, optimizing therapeutic regimens, and developing more effective rehabilitation strategies.

## 1 Background

Ramsay Hunt Syndrome (RHS), also known as herpes zoster oticus or geniculate ganglion syndrome, is an acute inflammatory disease caused by the reactivation of varicella-zoster virus (VZV) that invades the geniculate ganglion of the facial nerve (1). It is classically characterized by the triad of acute facial palsy, otalgia, and herpetic eruptions in the auricular region. Beyond this classic presentation, a substantial proportion of cases (approximately 30–50%) involve multiple cranial neuropathies, extending the clinical spectrum to include debilitating symptoms such as hearing loss, vertigo, dysphagia, and ophthalmoplegia. This multisystem involvement underscores RHS not merely as a disorder of the facial nerve, but as a complex polycranial neuritis with potential for severe neurological morbidity (2).

While the epidemiology, pathogenesis, and management of RHS-associated facial palsy are well-documented, the literature on the concomitant involvement of other cranial nerves remains notably fragmented (3). Existing knowledge is dispersed across isolated case reports and small series, lacking a cohesive synthesis. Critical questions regarding the exact incidence, anatomical mechanisms of spread (e.g., retrograde axonal versus hematogenous), characteristic clinical timelines, and differential prognosis for each cranial nerve remain inadequately addressed. This gap hinders a unified clinical understanding, potentially leading to diagnostic delays, especially in atypical or zoster sine herpete presentations (4), and suboptimal management strategies.

Therefore, a comprehensive review focusing specifically on cranial nerve involvement in RHS is urgently needed. This review aims to consolidate the current evidence on the epidemiology, clinical features, diagnostic approaches, and outcomes associated with the involvement of each cranial nerve (I-XII) in RHS. By synthesizing this dispersed knowledge, we seek to provide clinicians with a structured framework for timely recognition, accurate differential diagnosis, and informed prognostication of this complex neurological syndrome, ultimately guiding more effective patient care. A targeted literature search of the PubMed/MEDLINE database (from inception to December 2025) was conducted using the terms “Ramsay Hunt Syndrome,” “Varicella -zoster virus,” “Varicella zoster,” “neurological complication,” “neurological complications,” “cranial nerve,” “cranial nerves,” “polycranial” and “multiple cranial.”

## 2 Epidemiology, clinical features, and prognosis of cranial nerve involvement

### 2.1 RHS and inherent facial nerve (CN VII) involvement

RHS is an uncommon yet clinically recognizable entity with distinct epidemiological patterns, exhibiting an annual global incidence of approximately 5 cases per 100,000 population (5). It accounts for 16% of all causes of unilateral facial palsies in children and 18% in adults (1, 6)—ranking among the most prevalent non-traumatic causes of facial nerve paralysis—while its prevalence remains lower than that of Bell’s palsy (1). While RHS has been reported across a broad age spectrum (3 months to 82 years) (7), the elderly population is disproportionately affected. Similar to herpes zoster in general, the incidence exhibits a clear positive correlation with age, peaking in individuals aged 50 years and older (8), which aligns with the progressive deterioration of T-cell-mediated immunity in older adults. The incidence of RHS has gradually decreased over time because of the development of the varicella-zoster vaccine and better health facilities. However, a growing trend of RHS onset in young adults has been increasingly recognized in recent years (7, 9). This trend is closely associated with lifestyle-related factors, such as chronic sleep deprivation, excessive work-related stress, and the subsequent immune suppression that arises from these conditions. There is no clear racial, gender or ethnic predisposition to RHS. Unlike Bell’s palsy, which may have a slight seasonal variation, RHS does not demonstrate a consistent seasonal pattern, as viral reactivation is not typically linked to seasonal triggers. The primary trigger for RHS is reduced VZV-specific cellular immunity. Well-documented risk factors include: advanced age, diabetes, hypertension, chronic kidney disease, human immunodeficiency virus (HIV) infection, malignancy, trauma, and prolonged psychological stress (10). These factors disrupt the immune surveillance that maintains VZV in latency, facilitating viral reactivation.

The clinical hallmark of RHS is the combination of acute facial nerve dysfunction and a vesicular rash in the auditory region. The presentation can range from incomplete forms to severe polycranial neuropathies. The classic triad includes acute ipsilateral peripheral facial palsy, otalgia (ear pain) and vesicular rash in the ear (10). Acute peripheral facial palsy is the most defining feature, resulting from inflammation and damage to the facial nerve (CN VII). The palsy is typically unilateral and can vary in severity from mild weakness to complete paralysis. It often leads to inability to close the eye, brow drooping, and flattening of the nasolabial fold. Intense, deep, or burning ear pain (otalgia) in the ear often precedes the paralysis and rash by several days. Painful vesicles appear on the pinna and external auditory canal, and may extend to the tympanic membrane or soft palate, forming a pathognomonic rash. In rare cases, viral spread causes CNS involvement (including meningitis (11), encephalitis (12), myelitis (13)) and multiple cranial neuritis (e.g., involvement of the 8^th^, 9^th^, 10^th^, or 11^th^ cranial nerves) (3)—as well as permanent sequelae such as persistent facial palsy (in 30% of patients), chronic pain, or irreversible hearing loss (1). The ensuing discussion will focus on these manifestations.

### 2.2 Olfactory and optic nerves (CN I, II) “involvement”

The geniculate ganglion represents the pathological epicenter of Ramsay Hunt Syndrome (RHS). Involvement of adjacent cranial nerves (CNs V, VIII, IX, X) is well documented and likely occurs through perineural spread or shared cerebrospinal fluid compartments. In contrast, the olfactory (CN I) and optic nerves (CN II) reside within distinct anatomical regions—the anterior and middle cranial fossae, respectively—and are both embryologically and anatomically remote from the geniculate ganglion. CN I consists of unmyelinated axons traversing the cribriform plate, while CN II is essentially a central nervous system (CNS) tract. Therefore, direct involvement of these nerves in a localized geniculate ganglionitis appears highly improbable. Nonetheless, given the neurotropic nature of VZV and its potential for widespread reactivation and CNS invasion, it remains essential to evaluate whether credible evidence links RHS to dysfunction of these sensory nerves, while clearly distinguishing between mechanisms of direct extension and indirect complications.

Anosmia is a common clinical manifestation of olfactory nerve involvement. Our systematic search reveals no case reports or series that describe isolated anosmia as a direct feature of uncomplicated RHS, and large clinical studies on RHS do not list anosmia among the common or rare symptoms (14–16). More significantly, anosmia occurring in the setting of VZV infection tends to be linked to complicated cases (17). Specifically, in VZV encephalitis, impaired smell or taste often arises as part of non-specific neurological manifestations—this is attributed to VZV-induced involvement of the olfactory bulbs and olfactory tracts on the ventral surface of the frontal lobe. Importantly, such olfactory or gustatory dysfunction is not a peripheral nerve complication of RHS itself (18).

Currently, there are no reliable, directly retrievable clinical reports specifically focusing on optic nerve involvement in RHS. However, several studies have provided insights that enhance our understanding and interpretation of this current situation. One study revealed that among patients with VZV infection, approximately 10–20% develop herpes zoster ophthalmicus (HZO); in contrast, optic neuropathy occurs in less than 1% of HZO cases (19). This finding suggests that the incidence of optic neuropathy is extremely low among all VZV-infected patients (including those with RHS). Another retrospective, multi-center case series of 37 patients with VZV infection involving the cranial nerves and CNS revealed that clinical involvement of the optic nerve was identified in 12 patients (32%), while radiological evidence of optic nerve involvement was detected in as many as 44% of cases (20). Such studies indicate that the rate of clinically symptomatic optic nerve involvement is significantly lower than the actual rate of radiologically confirmed involvement in real-world settings, implying the potential existence of underdiagnosis and underreporting for optic nerve’s involvement in RNS setting in clinical practice.

### 2.3 Ocular motor nerves (CN III, IV, VI) involvement

Although the exact incidence of Ramsay Hunt Syndrome (RHS) involving the ocular motor nerves (CN III, IV, and VI) remains undefined, data from a large retrospective study by Tsau et al. (21) offer valuable context. Among 330 patients with cranial nerve zoster, the oculomotor, trochlear, and abducens nerves were each affected in only 0.3% of cases. This is in stark contrast to the far more frequent involvement of the trigeminal (57.9%), facial (52.1%), and vestibulocochlear (20.0%) nerves (21). While precise epidemiological data are lacking due to sporadic reporting, certain demographic patterns can still be discerned from published case reports. Notably, there appears to be a male predominance among affected individuals, with cases spanning a broad age range from middle to older adulthood. Despite its rarity, ocular motor nerve involvement in RHS carries significant clinical implications, often indicating a more extensive and severe central nervous system infection that warrants heightened clinical attention.

The presentation extends beyond the classic RHS triad. Patients typically develop the characteristic facial palsy and herpetic rash, followed by symptoms of ophthalmoplegia. The involvement of oculomotor nerve (CN III) in RHS can present as ptosis, mydriasis (a dilated pupil that reacts poorly to light), and impaired eye movement (adduction, elevation, and depression). Liu et al. (22) and Benavente et al. (23) describe patients with oculomotor nerve injury, one with a dilated pupil and another as part of a wider polyneuritis. Abducens nerve (CN VI) involvement appears to be the most frequently reported based on existing case studies (24). It results in impaired lateral gaze and horizontal diplopia. This involvement often correlates with the spread of inflammation to the brainstem—such as the abducens nucleus—or to the petrous apex, potentially leading to Gradenigo’s syndrome (23, 25). Trochlear Nerve (CN IV)’s involvement causes vertical or torsional diplopia, exacerbated by looking down and in. The presence of ophthalmoplegia often signifies a more aggressive infection. It is frequently accompanied by other neurological complications such as: vestibulocochlear nerve dysfunction (hearing loss, vertigo) (4, 22), meningitis or meningoencephalitis (headache, fever, altered mentation) (4, 23, 24), CNS vasculopathy (4), and Myelitis (22).

The prognosis of RHS with oculomotor nerve involvement varies. In general, early treatment and mild involvement are associated with a better outcome (25). Intriguingly, the coexistence of ocular motor nerve involvement may complicate the recovery trajectory of facial paralysis and contribute to a more protracted rehabilitation course (21).

### 2.4 Trigeminal nerve (CN V) involvement

Accumulating clinical evidence—including case reports and imaging studies—confirms that RHS can complicate trigeminal nerve (CN V) involvement, manifesting as trigeminal herpes zoster and associated neurological deficits in addition to its typical manifestations. Trigeminal HZ, independently, accounts for ~10–20% of all HZ cases, with the ophthalmic branch (V1) being the most commonly involved division, followed by the maxillary (V2) and mandibular (V3) branches. The co-occurrence of RHS and trigeminal HZ is extremely rare, with no precise epidemiological data available due to its sporadic reporting. Most cases are documented in single-patient case reports (26–33), reflecting its low prevalence. In the reported cases of Ramsay Hunt Syndrome (RHS) with trigeminal involvement, over 80% involve patients aged 55 years or older, reflecting a notable association with advanced age. While isolated cases have been documented in immunocompetent individuals, the condition more frequently occurs in patients with compromised immune function. Such immunocompromised states include, but are not limited to, diabetes mellitus (34), underlying malignancies, HIV infection (35), and organ transplantation or long-term use of immunosuppressive medications (36)—all of which may impair the body’ s ability to control varicella-zoster virus (VZV) reactivation and dissemination.

The clinical presentation of this comorbidity is a combination of classic RHS symptoms and trigeminal nerve-specific deficits, often with a temporal sequence (trigeminal symptoms preceding or following RHS onset) (31). All cases exhibit core RHS features: ipsilateral facial palsy, otalgia, and auricular/otic vesicular eruptions, and trigeminal nerve-specific manifestations vary by the trigeminal branch involved, with prodromal pain often being the initial and most misleading feature. Atypical odontogenic pain—such as the “tooth-like pain” documented in a case reported in Gerodontology (2018) (26)—or facial neuropathic pain (characterized as stabbing or burning) is common during this prodromal phase, frequently leading to misdiagnosis as dental pathology (e.g., pulpitis or tooth abscess) before the appearance of characteristic vesicular eruptions. Once eruptions develop, they follow a dermatomal distribution corresponding to the involved trigeminal branch: for the ophthalmic branch (V1), vesicles occur on the forehead, periorbital skin, and cornea, with potential progression to sight-threatening complications like keratitis or uveitis (13); for the maxillary branch (V2), lesions manifest on the cheek, nasal ala, upper lip, and intraoral mucosa, including palatal ulcers (37); and for the mandibular branch (V3), vesicles appear on the lower lip, chin, mandibular gingiva, and tongue, with rare but severe complications such as tooth exfoliation and mandibular alveolar osteonecrosis (27, 30). Beyond cutaneous and mucosal signs, neurological deficits are also prevalent, ranging from hypoesthesia (reduced sensation) to hyperesthesia (increased sensitivity) in the affected trigeminal dermatome; in severe cases, brainstem involvement—specifically of the spinal trigeminal nucleus and tract—causes persistent pain or profound sensory loss, which can be radiologically confirmed by MRI hyperintensities in these regions, as illustrated in some cases (32, 38).

Two distinct temporal patterns of symptom onset have been observed in cases of RHS complicated by trigeminal herpes zoster (HZ). The first and more commonly reported pattern involves trigeminal HZ preceding the development of RHS: trigeminal-specific symptoms—such as facial pain or dermatomal vesicles—manifest initially, typically 5 to 14 days before RHS-related features (including otalgia and ipsilateral facial palsy) emerge (26, 27, 30, 32). The second pattern, simultaneous onset, is rare and characterized by the appearance of both trigeminal HZ and RHS symptoms within a narrow timeframe of 48 h (31).

It is important to note that the prodromal phase of this condition may involve atypical odontogenic or facial pain (26), which has been reported to lead to potential misdiagnosis—including unnecessary dental interventions such as root canals—before the onset of characteristic vesicular eruptions. Therefore, clinicians may consider VZV infection as a possible differential diagnosis in elderly or immunocompromised patients presenting with unexplained facial or dental pain, particularly when other typical causes of such pain have been ruled out. Magnetic Resonance Imaging (MRI) may play a critical role in confirming trigeminal or brainstem involvement, as T2-weighted and FLAIR (fluid attenuated inversion recovery) sequences have been observed to reveal hyperintensities in structures such as the trigeminal ganglion, spinal trigeminal tract, or facial nerve in some cases; additionally, gadolinium enhancement might highlight ganglionic inflammation, as supported by findings in select studies (38, 39).

Prognostically, available data suggest that timely diagnosis and early initiation of antiviral treatment may correlate with more favorable outcomes (40), including potentially better recovery of facial function and shorter duration of trigeminal pain—though these associations are based on limited case reports rather than large-scale studies (26). Immunocompetent patients have been observed to achieve full recovery in some instances (37, 41), while elderly or immunocompromised individuals may face a higher risk of persistent post-herpetic neuralnia (PHN) or incomplete facial palsy recovery, highlighting the need for individualized prognostic assessments.

### 2.5 Vestibulocochlear nerve (CN VIII) involvement

Involvement of the 8th cranial nerve (vestibulocochlear nerve, responsible for hearing and balance) is a most common complication of RHS, occurring in approximately 30–50% of all RHS cases (42, 43). This high prevalence reflects the anatomical proximity of the vestibulocochlear nerve to the geniculate ganglion (the primary site of VZV reactivation in RHS), facilitating viral spread via local ganglionic and transneural pathways (2, 44). Notably, the incidence of vestibulocochlear involvement in RHS exhibits the same age-related upward trend as RHS itself: it is rare in children (accounting for <5% of pediatric RHS cases) but increases sharply in adults aged ≥60 years, with a peak incidence of 65% in individuals aged 70–80 years (42). This correlation aligns with the age-dependent decline in VZV-specific cellular immunity, which not only triggers RHS but also exacerbates viral dissemination to adjacent cranial nerves like the 8th nerve (3).

Vestibulocochlear nerve involvement in RHS presents as two distinct symptom clusters, corresponding to the nerve’ s cochlear (hearing-related) and vestibular (balance-related) divisions. Sensorineural hearing loss (SNHL) is the most common cochlear symptom, occurring in roughly 66–80% of RHS patients with 8th nerve involvement (42, 43). It is typically unilateral (ipsilateral to the facial palsy) and ranges in severity from mild (20–40 dB hearing loss) to profound (>80 dB). In 60% of cases, SNHL develops acutely—within 48 h of the onset of facial weakness or auricular vesicles—and progresses rapidly over 1–3 days (44). Tinnitus is present in 75% of patients with cochlear involvement, often described as a high-pitched, continuous “ringing” or “buzzing” sound ipsilateral to the affected ear (45). Tinnitus typically precedes SNHL by 12–24 h and may persist even if hearing recovers, particularly in elderly patients (46). Paroxysmal vertigo occurs in 65% of RHS patients with 8th nerve involvement, characterized by sudden, severe spinning sensations (vertigo) lasting minutes to hours, often accompanied by nausea, vomiting, and diaphoresis (13). Unlike benign paroxysmal positional vertigo (BPPV), vertigo in RHS is not triggered by head movements but is constant or recurrent, reflecting direct viral injury to the vestibular labyrinth or nerve. Gait instability is present in 50% of patients with vestibular involvement, ranging from mild unsteadiness to severe ataxia requiring assistance with ambulation. This symptom correlates with the degree of vestibular dysfunction: patients with severe vertigo often exhibit more pronounced gait disturbances due to impaired vestibular-ocular and vestibular-spinal reflexes (46). Additionally, a spontaneous, horizontal-torsional nystagmus beating away from the affected ear is also a classic clinical sign in RHS patients with vestibular involvement (47).

A key clinical feature of eighth nerve involvement in RHS is its close temporal relationship with the classic triad of peripheral facial palsy, auricular vesicles, and otalgia. Although large-scale prospective studies systematically documenting the exact timing are lacking, evidence from case reports allows the following observations. In approximately 70% of cases, vestibulocochlear symptoms (e.g., tinnitus, hearing loss, vertigo) emerge either concurrently with or within 72 h of facial palsy onset. This temporal profile is a useful distinguishing feature from other causes of facial palsy accompanied by eighth nerve dysfunction, such as acoustic neuroma or meningitis. In the remaining 30% of cases, eighth nerve symptoms may manifest after a delay of more than 3 days following the classic triad (48), which may lead to diagnostic delay if clinicians do not monitor for late-onset cranial nerve involvement in RHS patients (1).

In general, to confirm 8th cranial nerve involvement, three diagnostic tests are recommended by most scholars, including audiometry, vestibular function tests, and contrast-enhanced MRI of the brain and internal auditory canals. Pure-tone audiometry and speech audiometry are essential to document SNHL (a hallmark of cochlear nerve involvement), and a characteristic “downward-sloping” audiogram (worse hearing at high frequencies) is seen in 60% of cases (49). Vestibular function tests in relevant clinical reports mainly refers to caloric testing, video head impulse testing (vHIT) and vestibular evoked myogenic potentials (VEMPs) elicited by click sound (cVEMP) or galvanic stimulation (gVEMP): caloric testing (to assess horizontal semicircular canal function) often shows ipsilateral hypofunction (reduced or absent response to cold/warm water irrigation) in patients with vertigo or gait instability (50); vHIT may reveal impaired vestibular-ocular reflex (VOR) gain in the affected ear, reflecting dysfunction of the semicircular canals and their afferent pathways (44); cVEMP and gVEMP are utilized to evaluate the functional integrity of the lateral semicircular canal, saccule, and their respective afferent nerve fibers (50, 51). Collectively, the combination of these vestibular function tests enables precise localization of the lesion site responsible for vestibular dysfunction in RHS patients, thereby providing critical diagnostic insights for clinical management. Contrast-enhanced MRI of the brain and internal auditory canals may show enhancement of the 8th nerve or geniculate ganglion in 40–50% of cases, confirming viral inflammation (45). MRI also excludes alternative causes like acoustic neuroma or stroke.

The prognosis of vestibulocochlear dysfunction in RHS depends on three key factors: age, timing of treatment, and initial symptom severity (49). Overall, 50–60% of RHS patients with SNHL experience partial or complete hearing recovery, with favorable prognostic factors including young age (<60 years), early antiviral treatment and mild initial SNHL (<35 dB) (52). Tinnitus persists in 40% of patients, even if hearing recovers. Chronic tinnitus is more common in elderly patients and those with delayed treatment. Vestibular symptoms improve more rapidly than cochlear symptoms: 70% of patients experience resolution of vertigo within 2–4 weeks, and 85% regain normal gait within 1–2 months (53). This is due to central vestibular compensation (the brain adapting to unilateral vestibular loss) (13). Only 10% of patients have persistent vestibular symptoms (e.g., mild unsteadiness in dark environments), primarily those with severe initial vestibular hypofunction or delayed treatment. In comparison with RHS patients without 8th nerve involvement, those with 8th nerve involvement have a worse overall prognosis (49), with a higher rate of permanent facial palsy (35% vs. 20%) and increased risk of chronic pain (e.g., postherpetic neuralgia) (25% vs. 15%) (54).

### 2.6 Glossopharyngeal and vagus nerves (CN IX, X) involvement

Involvement of the 9th (glossopharyngeal) and 10th (vagus) cranial nerves represents an uncommon but clinically significant complication of RHS, occurring in 1.5–9% of all RHS cases (55, 56)—a rate that contrasts sharply with the 30–50% prevalence of 8th cranial nerve involvement in RHS. Notably, when the 9th and 10th nerves are involved, concurrent involvement of both nerves is the predominant pattern (55, 57–59); this phenomenon is primarily driven by their shared anatomical pathways (e.g., co-localization within the jugular foramen) and the sequential spread patterns of VZV (60). In contrast, isolated involvement of either the 9th or 10th nerve is extremely rare, accounting for <5% of RHS cases with lower cranial neuropathy. Notably, 9th and 10th nerve co-involvement is also rarely isolated: 82% of affected patients present with concurrent involvement of other cranial nerves (most commonly 8th), consistent with the “polyneuropathic pattern” of severe VZV reactivation (61). Population-based studies further demonstrate an age-related increase in risk: patients aged ≥65 years account for most of cases (58, 61), with a peak incidence in adults ≥80 years.

Clinical manifestations of 9th (glossopharyngeal) and 10th (vagus) cranial nerve involvement in RHS depend on the extent and severity of nerve damage. The 9th cranial nerve mediates pharyngeal sensation, taste from the posterior one-third of the tongue, and parotid gland secretion; its involvement typically presents with four key symptoms: odynophagia and dysphagia (the most common, affecting above 90% of cases), characterized by sharp pain with swallowing and difficulty initiating swallowing (55); impaired pharyngeal sensation (the second most common), often manifesting as a reduced ipsilateral gag reflex and increased aspiration risk; dysgeusia, defined as loss of taste in the posterior tongue, which is frequently overlooked due to more prominent swallowing symptoms; glossopharyngeal neuralgia, a rarest but severe symptom involving paroxysmal pain in the tonsillar fossa, triggered by swallowing or talking. Vagus nerve involvement primarily affects laryngeal and pharyngeal motor function: hoarseness is present in most of cases, resulting from paralysis of the ipsilateral vocal fold (confirmed by laryngoscopy in all reported cases); severe dysphagia and aspiration occur in three-quarters of patients, due to impaired coordination of pharyngeal constriction and palatal elevation, and partially require temporary nasogastric tube feeding; cardiac arrhythmias is rare but life-threatening, including bradycardia and atrioventricular block, secondary to parasympathetic fiber involvement; palatal palsy—manifested as ipsilateral uvular deviation—is identified in some RHS patients with 10th cranial nerve involvement, and this finding serves as a pathognomonic sign of vagal neuropathy.

A key temporal feature of 9th/10th nerve involvement in RHS is its delayed onset relative to the classic triad (peripheral facial palsy, auricular vesicles, auricular pain): roughly 80% of patients develop swallowing/voice symptoms 3–10 days after facial palsy (vs. the 72-h window for 8th nerve involvement), reflecting retrograde VZV spread from the geniculate ganglion to the jugular foramen (62). Notably, approximately 10% of patients present with atypical manifestations, where 9th/10th cranial nerve symptoms (e.g., isolated hoarseness or dysphagia) precede the classic triad. This sequence leads to misdiagnosis as laryngitis or stroke (63, 64).

Confirming 9th/10th nerve involvement in RHS requires a multimodal approach to distinguish VZV-induced neuropathy from mimics (e.g., stroke, tumor, myasthenia gravis): laryngoscopy/pharyngoscopy documents vocal fold paralysis (10th nerve) and impaired pharyngeal movement (9th nerve); contrast-enhanced MRI of the brainstem/jugular foramen shows 9th/10th nerve/ganglion enhancement in 63% of cases, with DWI (diffusion-weighted imaging) hyperintensities indicating acute inflammation (60, 65); CSF VZV DNA detection by PCR or serum anti-VZV IgM elevation confirms active infection; and video fluoroscopic swallow study (VFSS) identifies aspiration in some patients with clinical dysphagia (66).

RHS with 9th and 10th cranial nerve involvement carries a less favorable prognosis than uncomplicated RHS, with a higher likelihood of delayed recovery and persistent symptoms. Early standardized treatment (antivirals+corticosteroids), close monitoring of swallowing/respiratory function, and targeted rehabilitation (e.g., speech therapy for dysphagia) are critical to optimizing outcomes and reducing long-term morbidity (3).

### 2.7 Accessory nerve (CN XI) involvement

Through literature review, we found that 11th cranial nerve (accessory nerve) involvement does occur in RHS (67), though it is extremely rare and almost exclusively presents as part of a “poly-cranial neuropathy pattern”—often coexisting with 9th/10th nerve dysfunction (jugular foramen syndrome) (10) due to the anatomical proximity of these nerves at the jugular foramen. The accessory nerve innervates the sternocleidomastoid and trapezius muscles, so its dysfunction presents with unilateral neck and shoulder weakness. RHS with 11th cranial nerve involvement shares essentially similar epidemiology and demographic features to RHS with 9th/10th cranial nerve involvement. Crucially, 11th nerve symptoms always appear after 9th/10th nerve symptoms. Notably, the presence of 11th cranial nerve involvement in RHS often indicates a severe disease state and a poor prognosis (64). However, neck and shoulder weakness (the key sign of 11th cranial nerve involvement) is the most overlooked physical finding in clinical practice. Therefore, it cannot be ruled out that 11th cranial nerve involvement in RHS patients may be underdiagnosed and underreported.

### 2.8 Hypoglossal nerve (CN XII) involvement

The 12th nerve involvement in RHS is extremely rare, even less common than 11th nerve involvement. This extreme rarity is attributed to the hypoglossal nerve’ s anatomical isolation: it originates from the hypoglossal nucleus in the medulla oblongata and exits the skull via the hypoglossal canal (separate from the jugular foramen where 9th/10th/11th nerves exit). For VZV to reach the 12th nerve, it requires either extensive retrograde spread from the jugular foramen to the medulla or hematogenous dissemination—both of which are uncommon in RHS. Notably, nearly all documented cases of RHS-associated 12th nerve involvement occur alongside 9th/10th/11th nerve dysfunction (i.e., “jugular foramen syndrome plus hypoglossal palsy”). The hypoglossal nerve innervates the intrinsic and extrinsic muscles of the tongue (except the palatoglossus), so its dysfunction presents with unilateral tongue weakness and deviation.

Epidemiologically, it is extremely rare, mostly occurring in patients with underlying conditions such as diabetes (34) or rheumatic diseases (55), with a higher prevalence in middle-aged and elderly individuals, and almost always presents as part of polycranial neuropathy—commonly coexisting with involvement of cranial nerves V, VII, VIII, IX, and X (55, 68). Notably, it typically occurs secondarily to other cranial nerve palsies (e.g., facial palsy, vestibulocochlear dysfunction) rather than as an initial symptom. Prognostically, while combination therapy with antiviral agents (acyclovir/valacyclovir) and steroids generally leads to satisfactory recovery of most cranial nerve functions (34), 12th nerve involvement is associated with relatively prolonged recovery and may require supportive interventions; additionally, compared to RHS without polycranial involvement, cases with 12th nerve involvement have lower overall recovery rates and a higher risk of residual symptoms (69), though complete recovery is still possible with early and standardized treatment.

**Figure 1:**
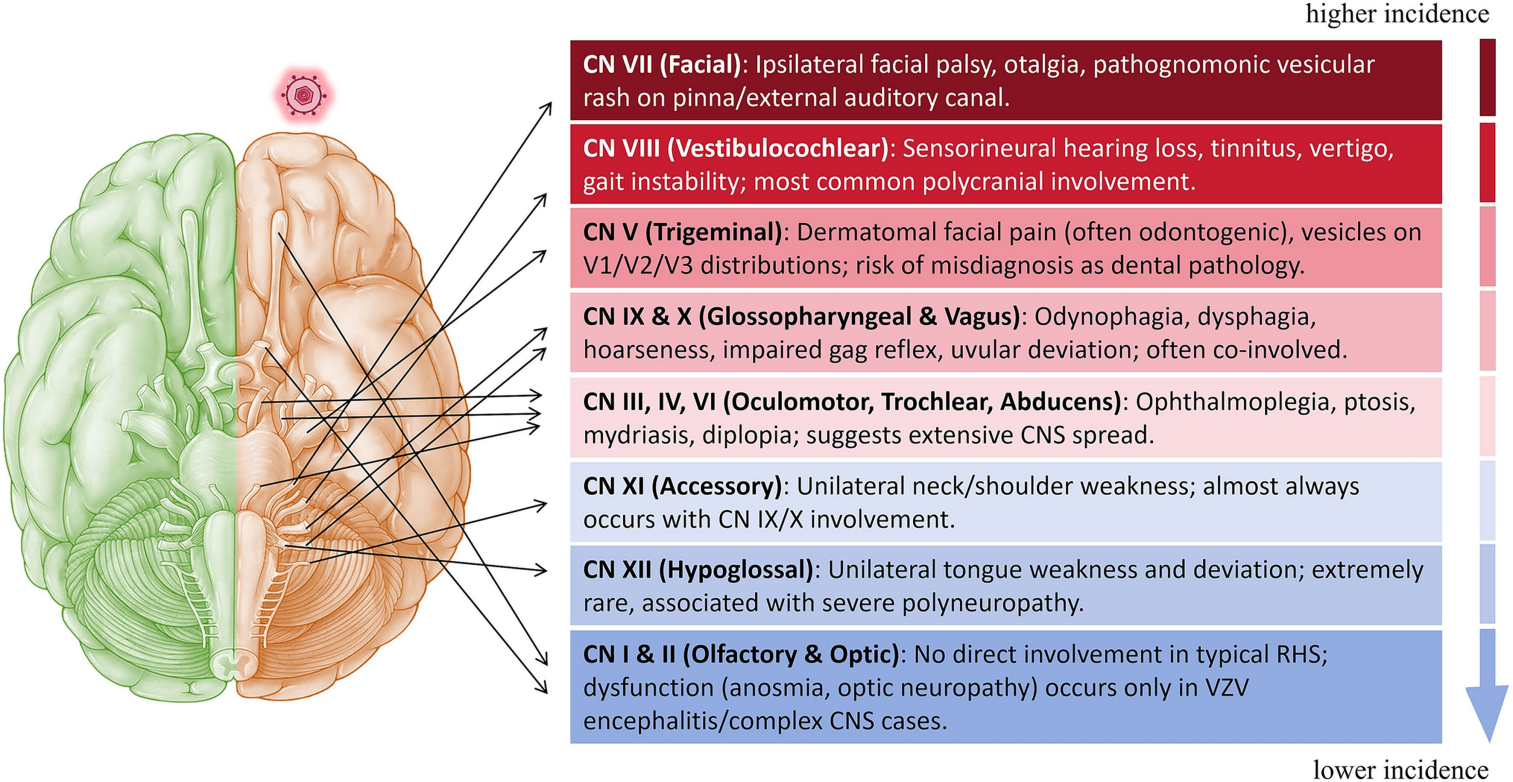
Schematic illustration summarizing the key clinical manifestations of cranial nerve involvement in Ramsay Hunt Syndrome (RHS). Cranial nerves are ordered by descending relative incidence.

Based on the detailed review above, cranial nerve involvement in RHS follows distinct patterns correlating with anatomical proximity to the geniculate ganglion. The vestibulocochlear nerve (CN VIII) is most frequently affected, while involvement of the trigeminal (CN V), glossopharyngeal/vagus (CN IX/X), and ocular motor nerves (CN III, IV, VI) signifies more extensive disease. Isolated accessory (CN XI) or hypoglossal (CN XII) nerve palsy is rare and typically occurs within a severe polycranial neuropathy. Direct involvement of the olfactory (CN I) and optic nerves (CN II) in uncomplicated RHS is highly unlikely. A schematic summary of the key clinical manifestations associated with each cranial nerve is provided in Figure 1, offering a visual overview to aid in clinical recognition.

## 3 Differential diagnosis of RHS with multiple cranial neuropathies

The presentation of multiple cranial neuropathies, as seen in RHS, necessitates a systematic diagnostic approach to distinguish it from a broad spectrum of infectious, inflammatory, neoplastic, and vascular conditions. RHS itself is characterized by the classic triad of acute peripheral facial palsy (CN VII), a vesicular rash in the ear or mouth, and frequent audiovestibular symptoms (CN VIII). Its acute onset, association with varicella-zoster virus (VZV), and generally self-limiting course are key identifiers. However, the absence of a rash (zoster sine herpete) or atypical features should prompt a rigorous search for mimics. The summary of differential diagnostic approach for RHS with multiple cranial neuropathies is seen in Table 1.

**Table 1:** Summary of differential diagnostic approach for RHS with multiple cranial neuropathies.

Diagnosis	Prevalence	Age distribution	Sex predilection	Mode of onset	Disease course	Key distinguishing features	Key diagnostic tests
RHS with multiple cranial neuropathies	Uncommon	Middle-aged adults (40–60 years); rare in children	No	Acute; precedes or coincides with vesicular rash	Self-limited with gradual recovery (4–12 weeks); residual deficits in ~30% of cases	• Peripheral facial palsy (CN VII) • Vesicular rash in ear/mouth (zoster) • CN VIII symptoms common • Other cranial nerves (V, IX, X) may be involved	• Clinical examination • PCR for VZV from vesicular fluid
Lyme disease	Common in endemic regions (e.g., North America, Europe)	Double peak in children (5–14 years) and adults (40–59 years)	No	Subacute (onset 1–4 weeks after tick bite); may follow erythema migrans	Resolves completely with timely antibiotic treatment (2–4 weeks); untreated cases may have relapsing or chronic symptoms	• Bilateral facial palsies are classic • History of tick bite, erythema migrans rash • Radiculoneuritis, meningitic symptoms • Endemic geographic area	• Serum & CSF serology (ELISA & Western Blot) • CSF lymphocytic pleocytosis
Sarcoidosis (neurosarcoid)	Rare (affects ~5–10% of all sarcoidosis patients)	Middle age (20–40 years); rare in children	Slight female predominance	Subacute to chronic (onset over weeks to months); may present with gradual neurological deficits	Chronic, relapsing–remitting course; responds to corticosteroids but often requires long-term maintenance therapy	• Basilar meningitis pattern • Other systemic involvement• Pituitary/hypothalamic dysfunction possible • Chronic, relapsing course	• Chest CT • Serum ACE (non-specific) • Tissue biopsy (e.g., lymph node) for non-caseating granulomas
GBS & its variants	Rare (incidence ~1–2 cases per 100,000 annually)	All ages; peak in elderly (>65 years) and children (1–5 years); MFS more common in adults	No	Acute (onset within hours to 1 week); often follows upper respiratory or gastrointestinal infection	Monophasic course; progressive weakness peaks at 2–4 weeks, then gradual recovery (weeks to months); rare residual disability	• Ascending, symmetric weakness & areflexia • MFS variant: triad of ophthalmoplegia, ataxia, areflexia • Can cause bilateral CN palsies (e.g., VII, IX, X)	• Clinical pattern & CSF albuminocytologic dissociation (high protein, normal cells) • Nerve conduction studies/EMG • Anti-GQ1b antibodies (for MFS)
Sjögren’s syndrome	Rare (neuro-Sjögren affects ~10–20% of Sjögren’s patients)	Middle-aged to elderly (40–60 years)	Marked female predominance (female:male ratio ~9:1)	Subacute to chronic (onset over weeks to months); may be insidious	Chronic, progressive or relapsing course; responds to immunosuppressive therapy (e.g., hydroxychloroquine, rituximab)	• Sicca symptoms (dry eyes, dry mouth) • Sensory ganglionopathy/peripheral neuropathy • Other autoimmune features • CNS manifestations possible	• Anti-SSA (Ro)/Anti-SSB (La) antibodies • Schirmer’s test, lip biopsy • Rheumatologic serology (ANA, RF)
Schwannoma	Rare (incidence ~1–2 cases per 100,000 annually; vestibular schwannoma is most common type)	Middle age (30–60 years); rare in children (except neurofibromatosis type 2)	Sight female predominance in vestibular schwannoma	Insidious (onset over months to years); gradual progression of symptoms	Slow, progressive course; benign tumor growth; symptoms worsen with increasing tumor size unless surgically resected	• Slowly progressive deficits • Vestibular schwannoma: unilateral SNHL, tinnitus (CN VIII); CN VII palsy is a late sign • Trigeminal schwannoma: facial numbness/pain	• MRI with contrast (IAC protocol) is gold standard
Meningioma	Uncommon (incidence ~2–3 cases per 100,000 annually)	Elderly (50–70 years); rare in children	Marked female predominance (female:male ratio ~2:1)	Insidious (onset over months to years); symptoms develop gradually due to slow tumor growth	Slow, progressive course; most are benign (atypical/malignant meningiomas have faster progression)	• Slow progression • Location-dependent symptoms (e.g., sphenoid wing, cerebellopontine angle) • May have hyperostosis of adjacent bone	• MRI with contrast (shows dural tail sign)
Skull base metastases	Rare (accounts for ~5–10% of all brain metastases)	Elderly (50–70 years); correlates with age of primary cancer	Dependent on primary cancer	Subacute (onset over weeks to months); may be abrupt if metastasis causes hemorrhage or edema	Progressive course; poor prognosis (median survival ~3–6 months); symptoms worsen with tumor growth or leptomeningeal spread	• Progressive, painful multiple cranial neuropathies • Known primary malignancy (e.g., breast, lung, melanoma) • Encephalopathy, radiculopathies	• Contrast-enhanced MRI (may show nodular enhancement) • CSF cytology (often requires high volume, repeated taps)
Carcinomatous meningitis	Rare (affects ~3–5% of cancer patients)	Elderly (50–70 years); linked to age of primary malignancy	Dependent on primary cancer	Subacute to acute (onset over days to weeks); rapid progression of neurological deficits	Aggressive, rapidly progressive course; poor prognosis (median survival ~1–3 months) despite chemotherapy	• Progressive, painful multiple cranial neuropathies • Known primary malignancy (e.g., breast, lung, melanoma) • Encephalopathy, radiculopathies	• Contrast-enhanced MRI (may show nodular enhancement) • CSF cytology (often requires high volume, repeated taps)
Brainstem stroke	Uncommon (accounts for ~10–15% of all ischemic strokes)	Elderly (>65 years); risk increases with age; rare in young adults	Slight male predominance	Acute (onset within seconds to minutes); “sudden onset” of neurological deficits (e.g., facial palsy, ataxia)	Monophasic course; recovery begins within days to weeks; residual deficits common (depends on stroke severity and location)	• Sudden, acute onset (minutes to hours) • “Crossed” signs (ipsilateral CN palsy + contralateral motor/sensory deficit) • Associated with vascular risk factors (HTN, DM, AFib)	• Diffusion-weighted MRI (most sensitive for acute ischemia) • CT angiogram/MR angiogram
Gradenigo’s syndrome	Extremely rare (classic triad: otitis media, abducens palsy, facial pain)	Children and young adults (5–25 years); rare in elderly	No	Acute (onset within days of untreated otitis media); symptoms worsen rapidly without antibiotics	Resolves with prompt antibiotic treatment (4–6 weeks); untreated cases may lead to intracranial complications (abscess, meningitis)	• Classic triad: Otitis media + Facial pain (CN V) + Abducens palsy (CN VI) • Due to petrous apicitis	• CT/MRI of temporal bone showing petrous apex inflammation • Clinical history of otitis media
Idiopathic inflammatory pseudotumor (Tolosa-Hunt Syndrome)	Extremely rare (incidence <0.1 cases per 100,000 annually)	Middle age (40–60 years); rare in children	No	Acute to subacute (onset over days to weeks); severe orbital pain precedes cranial nerve palsy (CN III, IV, VI)	Relapsing–remitting course; rapid response to high-dose corticosteroids (symptoms improve within 72 h); long-term maintenance to prevent relapses	• Painful ophthalmoplegia (CN III, IV, VI ± V1) • Symptoms may resolve spontaneously and relapse • Excellent response to corticosteroids	• MRI (may show cavernous sinus enhancement) • Diagnosis of exclusion after ruling out neoplasms/aneurysms
Diabetic cranial neuropathy	Uncommon (affects ~1–2% of diabetic patients annually)	Middle-aged to elderly (50–70 years); risk increases with duration of diabetes	No	Acute (onset within hours to days); often painless (unlike ischemic neuropathy)	Monophasic course; spontaneous recovery within 2–6 months (90% of cases); rare residual deficits (more common in long-standing, poorly controlled diabetes)	• Typically a mononeuropathy (e.g., CN III) • CN III palsy is usually pupil-sparing • Acute/subacute onset • Presence of long-standing or poorly controlled diabetes	• Diagnosis of exclusion • HbA1c, blood glucose • Must rule out other compressive/ischemic causes with MRI/MRA if atypical

RHS, Ramsay Hunt Syndrome; CV, cranial nerve; PCR, polymerase chain reaction; VZV, varicella-zoster virus; CSF, cerebrospinal fluid; ELISA, enzyme-linked immunosorbent assay; CT, computed tomography; ACE, angiotensin-converting enzyme; GQ1b, ganglioside Q1b; Anti-SSA (Ro), Anti-Sjögren’s-syndrome-related antigen A; Anti-SSA (La), Anti-Sjögren’s- syndrome-related antigen B; ANA, antinuclear antibodies; IAC: internal auditory canal; SNHL, sensorineural hearing loss; MRI, magnetic resonance imaging; MRA, magnetic resonance angiography; HTN, hypertension; DM, diabetes mellitus; AFib, atrial fibrillation.

Among infectious causes, Lyme neuroborreliosis is a prime consideration, particularly in endemic areas. Its subacute onset weeks after a tick bite, potential for bilateral facial palsies, and accompanying meningitic symptoms help differentiate it from RHS (70). Serological testing of serum and CSF is diagnostic. Inflammatory disorders present with more subacute to chronic courses. Neurosarcoidosis often involves a basilar meningitis pattern and is typically accompanied by systemic findings like hilar lymphadenopathy (71). Guillain-Barré Syndrome (GBS) and its Miller Fisher Syndrome variant cause acute, ascending, and often bilateral neuropathies, with CSF showing albuminocytologic dissociation (72).

Neoplastic processes, such as schwannoma (73), meningioma (74), and skull base metastases (75), are critical “cannot-miss” diagnoses. They are distinguished by their insidious, progressive, and often painful course. A history of known malignancy raises suspicion for metastases or carcinomatous meningitis, the latter being confirmed via repeated CSF cytology. Vascular events, notably brainstem stroke, present with the most acute, sudden-onset deficits and often exhibit “crossed” neurological signs, with diffusion-weighted MRI being the diagnostic cornerstone.

Finally, rare but classic syndromes must be considered. Gradenigo’s Syndrome presents with the triad of otitis media, facial pain (CN V), and abducens palsy (CN VI) (76). Tolosa-Hunt Syndrome is characterized by painful ophthalmoplegia with a dramatic response to corticosteroids (77).

In summary, the diagnostic workup for a patient presenting with a RHS-like picture should be guided by the onset, course, and pattern of cranial nerve involvement. Acute presentations favor RHS, Lyme, GBS, or stroke. Subacute or progressive courses demand investigation for inflammatory or neoplastic causes. Essential initial steps include a detailed history, contrast-enhanced MRI of the brain, and lumbar puncture with CSF analysis, followed by targeted serological and systemic testing based on the initial findings.

## 4 Mechanisms of cranial nerve involvement in RHS

**Figure 2:**
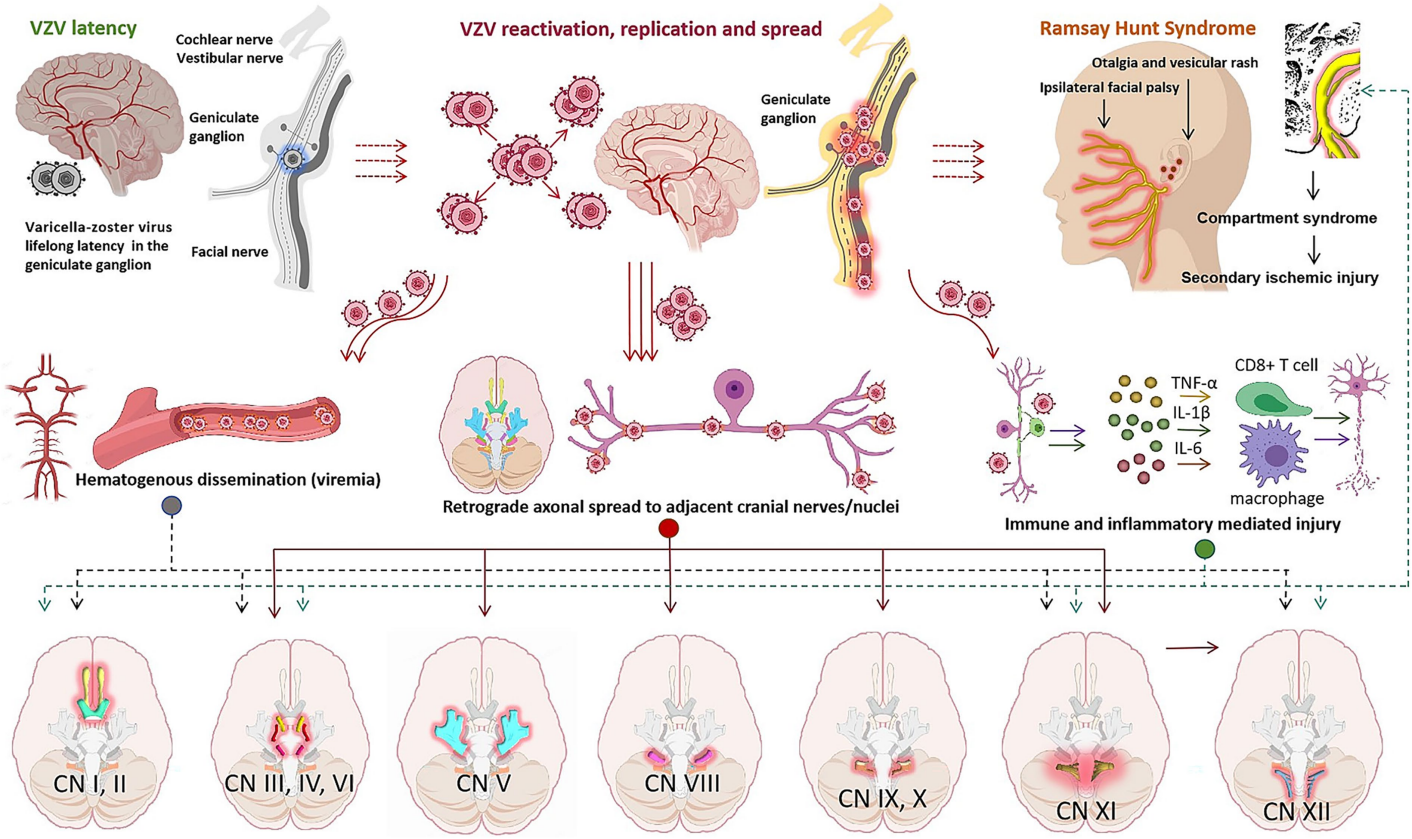
Mechanisms of cranial nerve involvement in RHS. RHS-related cranial neuropathy stems from VZV reactivation (latent in geniculate ganglia post-chickenpox) and spread, modulated by host immunity. VZV reactivates under immunosuppression, then spreads via retrograde axons (to CN VIII/V/VI/IX/X/XI/XII, causing sequential symptoms) or rare hematogenous dissemination (viremia, distant nerve involvement—CN I/II/III/IV/XI/XII—in immunocompromised patients). Viral replication triggers pro-inflammatory responses (cytokines, immune cells), leading to demyelination, axonal degeneration, and facial canal “compartment syndrome”—causing more severe nerve dysfunction than Bell’s palsy.

RHS-associated cranial neuropathy arises from VZV reactivation and spread, with severity modulated by host immunity and anatomical proximity to the primary reactivation site. The underlying mechanisms are multifactorial and can be summarized in three interconnected dimensions (Figure 2).

### 4.1 VZV reactivation in sensory ganglia

Following primary VZV infection (chickenpox), the virus undergoes retrograde axonal transport to establish lifelong latency in sensory ganglia. In the case of RHS, this latent infection resides specifically within the geniculate ganglion. Upon cellular immunosuppression (e.g., due to aging, stress, illness, or co-infection), latent VZV reactivates and begins replicating within the geniculate ganglion neurons (3), which serves as the inciting event for all subsequent pathways in this syndrome.

### 4.2 Pathways of cranial nerve spread

VZV spreads via two primary routes to involve additional cranial nerves, explaining the sequential symptom onset in clinical cases. First, retrograde axonal spread (most common): VZV travels along nerve axons from the geniculate ganglion to adjacent cranial nerves/nuclei via anatomical connections—CN VII to CN VIII (via direct anastomoses in the internal auditory canal, causing hearing loss/vertigo) (46), CN VII to CN V (via geniculate ganglion anastomoses, leading to trigeminal neuritis with otalgia/facial numbness) (78), CN VII to jugular foramen nerves (CN IX/X/XI, via nervus intermedius retrograde spread, causing dysphagia/hoarseness/neck weakness) (60), and jugular foramen to CN XII (further retrograde spread to medullary hypoglossal nucleus via shared brainstem pathways, explaining delayed CN XII symptoms) (34). Second, hematogenous dissemination (rare, severe): VZV enters the bloodstream (viremia) to seed distant cranial nerve nuclei/peripheral nerves, typically in immunocompromised patients (35, 36). This explains anatomically isolated nerve involvement (e.g., CN XII exiting via hypoglossal canal) and is associated with brainstem encephalitis, where MRI DWI hyperintensities in cranial nerve nuclei confirm central spread.

### 4.3 Immune and inflammatory mediated injury

Viral replication triggers a robust local immune response that significantly contributes to nerve damage: Infected cells release pro-inflammatory cytokines (TNF-*α*, IL-1β, IL-6), recruiting cellular immune effectors (T-cells, macrophages) and causing demyelination and axonal degeneration (1). The resulting inflammation leads to severe edema within the confined bony facial canal, creating a “compartment syndrome” that compresses the nerve and its microvasculature, leading to secondary ischemic injury—this mechanism explains why RHS often causes more severe and less reversible nerve dysfunction than Bell’s palsy (16).

## 5 Research prospects

Despite advancements in understanding RHS complicated with cranial nerve injury, current research still has limitations, and future studies can focus on the following key directions: to further explore the molecular mechanisms underlying VZV invasion of cranial nerves, clarify the role of inflammatory mediators in nerve injury, and thereby provide a theoretical basis for targeted therapy; to conduct multi-center, large-sample randomized controlled trials to compare the efficacy of different antiviral drug doses and glucocorticoid administration regimens, while exploring the application value of immunomodulators (e.g., intravenous human immunoglobulin) in severe cases; to integrate clinical indicators, imaging features, and laboratory test results to establish individualized prognostic prediction models that can guide clinical treatment decisions; and to develop innovative rehabilitation technologies (such as transcranial magnetic stimulation and virtual reality rehabilitation training) to enhance the recovery of cranial nerve function and improve patients’ quality of life.

## 6 Conclusion

Herein, we reviewed the epidemiological characteristics, clinical features, prognosis, and potential mechanisms of cranial nerve involvement in patients with RHS, aiming to provide valuable clinical information and insights for basic research. By methodically organizing the clinical manifestations associated with different cranial nerve involvements, this review aims to help clinicians rapidly identify the type of cranial nerve injury based on presenting symptoms, thereby reducing misdiagnosis rates. The summary of prognostic factors enables clinicians to assess the patient’s recovery potential early, formulate individualized follow-up and rehabilitation plans, and improve the patient’s quality of life. From the perspective of academic research, this review provides a clear research context for scholars in the field of RHS and cranial nerve involvement. By summarizing the shortcomings of current research (such as the unclear molecular mechanism of VZV invading cranial nerves and the lack of large-sample randomized controlled trials for treatment regimens), it points out four key research directions (pathogenesis exploration, treatment regimen optimization, prognostic prediction model construction, and rehabilitation technology innovation). This not only helps new researchers quickly grasp the core issues of the field but also provides a basis for experienced scholars to determine future research priorities, promoting the development of related disciplines.

## Glossary

RHS: Ramsay Hunt Syndrome
VZV: Varicella-zoster virus
CN: Cranial Nerve
HIV: Human immunodeficiency virus
HZO: Herpes zoster ophthalmicus
CNS: Central nervous system
HZ: Herpes zoster
MRI: Magnetic resonance imaging
FLAIR: Fluid attenuated inversion recovery
PHN: Post-herpetic neuralnia
SNHL: Sensorineural hearing loss
BPPV: Benign paroxysmal positional vertigo
vHIT: Video head impulse testing
VEMPs: vestibular evoked myogenic potentials
cVEMP: Cervical Vestibular Evoked Myogenic Potential
gVEMP: Genital Vestibular Evoked Myogenic Potential
VOR: Vestibular-ocular reflex
DWI: Diffusion-weighted imaging
VFSS: Video fluoroscopic swallow study
DNA: Deoxyribonucleic acid
TNF-*α*: Tumor necrosis factor-αlpha
IL-1β: Interleukin-1 βeta
IL-6: Interleukin-6
GBS: Guillain-Barré Syndrome
PCR: polymerase chain reaction
ELISA: enzyme-linked immunosorbent assay
ACE: angiotensin-converting enzyme
GQ1b: ganglioside Q1b
Anti-SSA (Ro): Anti-Sjögren’s-syndrome-related antigen A
Anti-SSA (La): Anti-Sjögren’s- syndrome-related antigen B
ANA: antinuclear antibodies
IAC: internal auditory canal protocol
HTN: hypertension
DM: diabetes mellitus
AFib: atrial fibrillation
